# Noninvasive prediction of Ki-67 expression level in IDH-wildtype glioblastoma using MRI histogram analysis: comparison and combination of MRI morphological features

**DOI:** 10.3389/fonc.2025.1577816

**Published:** 2025-05-16

**Authors:** Qiang Liang, Qiang Li, Xianwang Liu, Shuqi Shao, Yawen Pan, Hongyu Wang

**Affiliations:** ^1^ Department of Neurosurgery, The Second Hospital & Clinical Medical School, Lanzhou University, Lanzhou, Gansu, China; ^2^ Key Laboratory of Neurology of Gansu Province, Lanzhou University, Lanzhou, Gansu, China; ^3^ Academician Workstation of The Second Hospital & Clinical Medical School, Lanzhou University, Lanzhou, Gansu, China; ^4^ The Second Hospital & Clinical Medical School, Lanzhou University, Lanzhou, Gansu, China

**Keywords:** brain tumor, glioblastoma, magnetic resonance imaging, histogram analysis, Ki-67

## Abstract

**Purpose:**

To assess and compare the effectiveness of magnetic resonance imaging (MRI) morphological features and MRI histogram analysis in noninvasively predicting Ki-67 expression levels in patients with IDH-wildtype glioblastoma.

**Methods:**

Forty-six cases of IDH-wildtype glioblastoma with measured Ki-67 expression levels from January 2022 to July 2024 were retrospectively collected. They were divided into Ki-67 low-level expression group (Ki-67<20%, n=20) and Ki-67 high-level expression group (Ki-67≥20%, n=26) according to Ki-67 expression level. MRI morphological features were assessed and recorded. MRI histogram analysis were performed on contrast-enhanced T1-weighted images. Differences between these parameters were compared between the two groups. The diagnostic performance was assessed by the area under the receiver operating characteristic curve (AUC). Spearman correlation was used to evaluate the relationship between histogram parameters and Ki-67 expression level.

**Results:**

Hemorrhage was more prone to occur in the Ki-67 high-level expression group (*P*=0.017). The min, P01, P50, and P75 of IDH-wildtype glioblastoma Ki-67 high-level expression group were higher than those of the Ki-67 low-level expression group (*P*<0.00357). There was a significant positive correlation between the min (*r*=0.774), P01 (*r*=0.729), P50 (*r*=0.625), P75 (*r*=0.591), and Ki-67 expression level (*P*<0.05). The optimal diagnostic performance was obtained by combining MRI morphological features and histogram parameters, with an AUC of 0.867.

**Conclusion:**

Both MRI morphological features and histogram parameters could predict the Ki-67 expression level in IDH-wildtype glioblastoma, and the combined model integrating MRI morphological features and histogram parameters can be an excellent imaging biomarker for noninvasively predicting Ki-67 expression levels in patients with IDH-wildtype glioblastoma.

## 1 Introduction

Gliomas are the most common intracranial primary malignant brain tumor among adults (1, 2). Isocitrate dehydrogenase (IDH)-wildtype glioblastoma belongs to the WHO grade 4 tumor and has a poor prognosis, with a 5-year survival rate of approximately 5.1% (3). Among the many reported biomarkers for IDH-wildtype glioblastoma, the Ki-67 expression level is the most recognized and widely used biological marker (4). Ki-67 is a nuclear antigen closely associated with the degree of proliferative activity of tumor cells, and high levels of Ki-67 expression indicate increased invasiveness of the tumor and a poor prognosis for patients (5, 6). Previous literature has reported that the overall survival rate of IDH-wildtype glioblastoma is significantly reduced when the Ki-67 expression level is higher than 20% (7, 8). Furthermore, some investigators believe that the Ki-67 expression level is a reliable indicator for assessing the aggressiveness of IDH-wildtype glioblastoma, with a higher Ki-67 expression level indicating a more aggressive tumor that may require more intense therapeutic interventions (6–8). However, the Ki-67 expression level can only be obtained by invasive methods such as biopsy or surgical specimens (9, 10). Therefore, accurately predicting the Ki-67 expression level in IDH-wildtype glioblastoma is clinically significant, as it helps to assess the biological behavior of the tumor and provides assistance in making patient treatment strategies.

Magnetic resonance imaging (MRI) is routinely used in the evaluation of brain tumors, and contrast-enhanced T1-weighted images are the most commonly used sequence (8, 11). Contrast-enhanced T1-weighted images are acquired following the administration of a contrast agent based on T1 images, which allows for clear visualization of both the boundaries and internal characteristics of the lesion. Several MRI morphological features obtained from contrast-enhanced T1-weighted images have been shown to correlate with Ki-67 expression levels in IDH-wildtype glioblastoma (8). However, these MRI morphological features are relatively subjective and may be challenging for junior physicians in primary care institutions to master. In addition, conventional MRI morphological features analysis underutilizes the vast amount of information related to tumor heterogeneity encapsulated in contrast-enhanced T1-weighted images. Histogram analysis, as an objective and reproducible image analysis method, can extract multiple quantitative information reflecting tumor heterogeneity from medical images (11–13). Numerous studies have demonstrated that contrast-enhanced T1-weighted images histogram analysis is of great significance in the diagnosis, differentiation, and biological behavioral assessment of various brain tumors (14–16).

Therefore, this study aims to assess and compare the effectiveness of MRI morphological features and histogram analysis in noninvasively predicting Ki-67 expression levels in patients with IDH-wildtype glioblastoma.

## 2 Materials and methods

### 2.1 Patients

**Figure 1:**
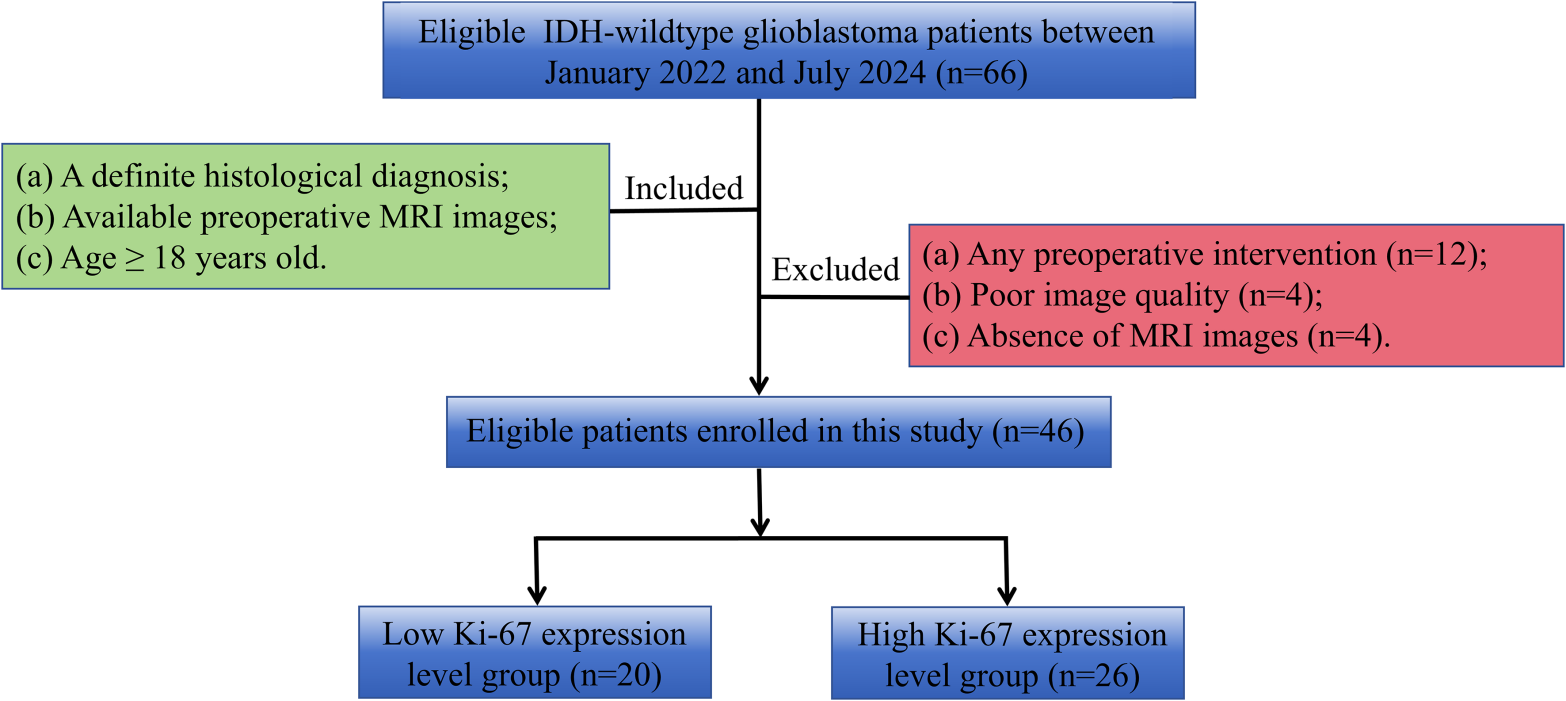
Flowchart of inclusion and exclusion criteria.

This retrospective study was approved by our Institutional Review Board, and the informed consent was waived. From January 2022 to July 2024, patients of IDH-wildtype glioblastoma with measured Ki-67 expression levels in Lanzhou University Second Hospital were collected. Inclusion criteria were: (1) patients with a definite histological diagnosis; (2) available preoperative MRI examination within two weeks before the operation; (3) age≥18 years. Exclusion criteria were: (1) any preoperative intervention; (2) motion artifacts or other factors that made the MRI images unable to be analyzed; (3) absence of contrast-enhanced T1-weighted images. The patient inclusion and exclusion flow chart is shown in **Figure 1**.

### 2.2 MRI examination

MRI examination was conducted using a 3.0-T MRI Scanner (Siemens Verio, Erlangen, Germany). The scan sequence and parameters were as follows: T1-weighted images: repetition time [TR]=250 ms, echo time [TE]=2.48 ms, slice thickness 5 mm, slice spacing 1.0 mm, the field of view 22 cm×22 cm, and matrix 256×256. T2-weighted images: TR=4000 ms, TE=96 ms, slice thickness 5 mm, slice spacing 1.0 mm, the field of view 22 cm×22 cm, and matrix 256×256. Contrast-enhanced T1-weighted images were obtained after injection of the contrast agent Gd-DTPA (0.1 mmol/kg) via the elbow vein hyperbaric mass at a flow rate of 3.0 ml/s, and the scanning parameters were the same as those of the T1-weighted images.

### 2.3 Image analysis

#### 2.3.1 MRI morphological features

Two radiologists with 10 and 18 years of experience in diagnostic neuroimaging reviewed and analyzed all the images, respectively. If a dispute occurs during the evaluation, agreement is reached through negotiation. MRI morphological features, including location (frontal, temporal, parietal, posterior fossa, or others), margin (fuzzy or clear), necrosis/cystic changes (no or yes), hemorrhage (no or yes), enhancement pattern (ring enhancement or non-ring enhancement), and the maximum diameter of tumor were assessed and recorded. Detailed definitions of MRI morphological features can be found in the literature (9).

### 2.4 Histogram analysis

**Figure 2:**
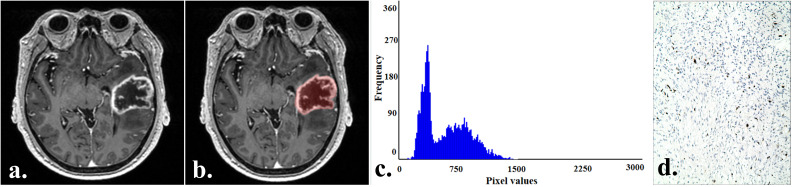
A 69-year-old man with an IDH−wildtype glioblastoma. **(a)** Contrast-enhanced T1-weighted image of the tumor. **(b)** Contrast-enhanced T1-weighted image with marked ROI of the tumor. **(c)** Histogram of the ROI, with min:123, P01:214, P50:516, and P75:762. **(d)** Immunohistochemistry showed the Ki-67 expression was about 15% (HC×100).

**Figure 3:**
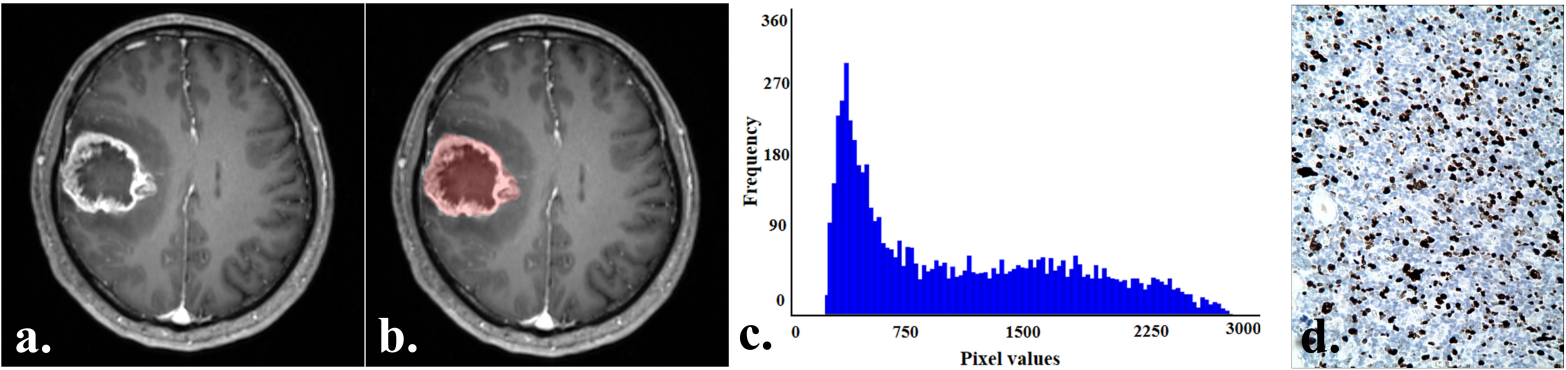
A 57-year-old man with an IDH−wildtype glioblastoma. **(a)** Contrast-enhanced T1-weighted image of the tumor. **(b)** Contrast-enhanced T1-weighted image with marked ROI of the tumor. **(c)** Histogram of the ROI, with min:226, P01:264, P50:912, and P75:1682. **(d)** Immunohistochemistry showed the Ki-67 expression was about 50% (HC×100).

Tumor sketching and histogram parameter extraction were performed using FireVoxel software (New York University, NY, USA; https://www.firevoxel.org/) on enhanced T1-weighted images by two radiologists who performed the MRI morphological features evaluation, respectively. Following previous literature (13), the largest tumor slice was first selected, and then the region of interest (ROI) encompassing all tumor components was sketched along the tumor edges. The software automatically extracted histogram parameters, including the minimum (min), maximum (max), mean, 1st (P01), 25th (P25), 50th (P50), 75th (P75), 99th (P90) percentile, standard deviation, variance, coefficient of variation, skewness, kurtosis, and entropy. Typical cases from the two groups are illustrated in **Figures 2** and **3**.

### 2.5 Pathological evaluation

A neuropathologist with 15 years of experience performed pathological evaluation based on the newest 2021 World Health Organization Classification of Tumors of the Central Nervous System (2). Ki-67 expression levels were stained and assessed using an anti-MIB-1 monoclonal antibody (DakoCytomation, Glostrup, Denmark). One thousand cells were counted in 10 randomly selected high magnification fields under 400× magnification, and the Ki-67 expression level was expressed as a percentage of the number of positive cells to the total number of cells. According to previous studies, a Ki-67 expression level <20% was defined as a Ki-67 low-level expression group, and a Ki-67 expression level ≥20% was defined as a Ki-67 high-level expression group (9, 10).

### 2.6 Statistical analysis

Statistical analysis were performed using MedCalc software (v. 19.1, Mariakerke, Belgium). The chi-square test was used to compare categorical variables. Intraclass correlation coefficient (ICC) was used to assess the inter-observer reproducibility of the histogram parameters (17). After the histogram parameters were tested for normality by the Shapiro-Wilk test, the independent t-test (normal distribution) or Mann-Whitney U-test (non-normal distribution) was used to compare the differences in the histogram parameters between the two groups. Binary logistic regression analysis was used to obtain a combined variable of the significant MRI morphological features and histogram parameters. Receiver operating characteristic (ROC) curves were conducted to determine the diagnostic performance. The Youden index was used to identify the optimal cut-off value from the ROC curve to maximize sensitivity, specificity, and accuracy. Delong’s test was used to compare the differences between AUCs. Spearman correlation analysis was used to assess the correlation between the histogram parameters and Ki-67 expression level. For 14 histogram parameters, multiple comparisons were performed using the Bonferroni correction, and a corrected *P*<0.00357 (0.05/14) was considered a significant difference. A *P*<0.05 was considered statistically significant.

## 3 Results

### 3.1 Clinical characteristics

Finally, 46 patients with IDH-wildtype glioblastoma were included in this study. Among the 46 patients diagnosed with IDH-wildtype glioblastoma, 20 cases were in the low-level expression group (Ki-67 expression level: 12.50 ± 3.93%), consisting of 6 males and 14 females, with an average age of 52.90 ± 9.50 years. Meanwhile, there were 26 cases in the high-level expression group (Ki-67 expression level: 45.96 ± 16.25%), consisting of 13 females and 13 males, with an average age of 54.85 ± 11.27 years. However, there were no significant differences in age (*P*=0.172) or sex (*P*=0.538) between the two groups.

### 3.2 Comparison of MRI morphological features between low- and high-level expression of Ki-67 in IDH-wildtype glioblastoma

Detailed MRI morphological features between low- and high-level expression of Ki-67 in IDH-wildtype glioblastoma were presented in **Table 1**. Hemorrhage was more prone to occur in the Ki-67 high-level expression group, compared with the Ki-67 low-level expression group (*P*=0.017). However, no differences were observed in other MRI morphological features between the two groups (*P*=0.072–0.741).

**Table 1:** Comparison of MRI morphological features between low- and high-level expression of Ki-67 in IDH-wildtype glioblastoma.

Parameters	Low-level expression group (n=20)	High-level expression group (n=26)	*p*
Location			0.174
frontal	10	12	
temporal	6	3	
parietal	2	5	
posterior fossa	1	0	
others	1	6	
Margin			0.515
fuzzy	12	18	
clear	8	8	
Haemorrhage			0.017
no	14	9	
yes	6	17	
Necrosis/cystic changes			0.741
no	6	9	
yes	14	17	
Across the midline			0.293
no	10	17	
yes	10	9	
Enhancement pattern			0.736
ring enhancement	9	13	
non-ring enhancement	11	13	
Maximum diameter (cm)	3.35 ± 0.96	3.89 ± 1.02	0.072

### 3.3 Comparison of histogram parameters between low- and high-level expression of Ki-67 in patients with IDH-wildtype glioblastoma

There was good agreement between the histogram parameters extracted by the two observers (ICCs:0.818-0.999, **Table 2**). The min, P01, P50, and P75 of the IDH-wildtype glioblastoma Ki-67 high-level expression group were higher than those of the Ki-67 low-level expression group (*P*<0.00357, **Table 2**). However, there was no significant difference between the two groups in max, mean, P25, P99, standard deviation, variance, coefficient of variation, skewness, kurtosis, or entropy (*P*>0.00357).

**Table 2:** Comparison of histogram parameters between low- and high-level expression of Ki-67 in IDH-wildtype glioblastoma.

Parameters	Low-level expression group (n=20)	High-level expression group (n=26)	*P*	ICC
Min	108.50 (70.50, 128.75)	187.50 (123.25, 323.75)	<0.001*	0.999
Max	875.50 (664.75, 1029.00)	960.00 (837.50, 1422.08)	0.068	0.994
Mean	377.86 (306.94, 449.24)	465.82 (328.15,650.56)	0.066	0.999
P01	149.50 (116.00, 276.00)	237.00 (162.50, 427.50)	<0.001*	0.998
P25	242.00 (201.00, 336.75)	408.50 (251.75, 638.25)	0.006	0.997
P50	334.50 (277.00, 410.25)	514.00 (407.50, 739.50)	0.003*	0.999
P75	475.00 (367.25, 596.75)	638.50 (528.25, 820.25)	0.002*	0.998
P99	681.00 (595.25, 681.00)	844.00 (720.50, 1231.00)	0.008	0.998
Standard deviation	123.17 (96.97, 190.39)	161.27 (126.41, 277.11)	0.035	0.999
Variance	16494.99 (11805.04, 36320.22)	19321.67 (14412.98, 52962.36)	0.278	0.983
Coefficient of variation	0.39 ± 0.11	0.35 ± 0.14	0.416	0.927
Skewness	0.33 ± 0.71	0.25 ± 0.69	0.683	0.998
Kurtosis	-0.44 (-0.63, 0.28)	-0.50 (-0.90, 0.09)	0.298	0.976
Entropy	4.11 ± 0.20	4.18 ± 0.21	0.330	0.818

ICC, intraclass correlation coefficient (ICC); *P value<0.00357(0.05/14), significant difference after Bonferroni correction for multiple comparisons.

### 3.4 Diagnostic performance analysis

**Figure 4:**
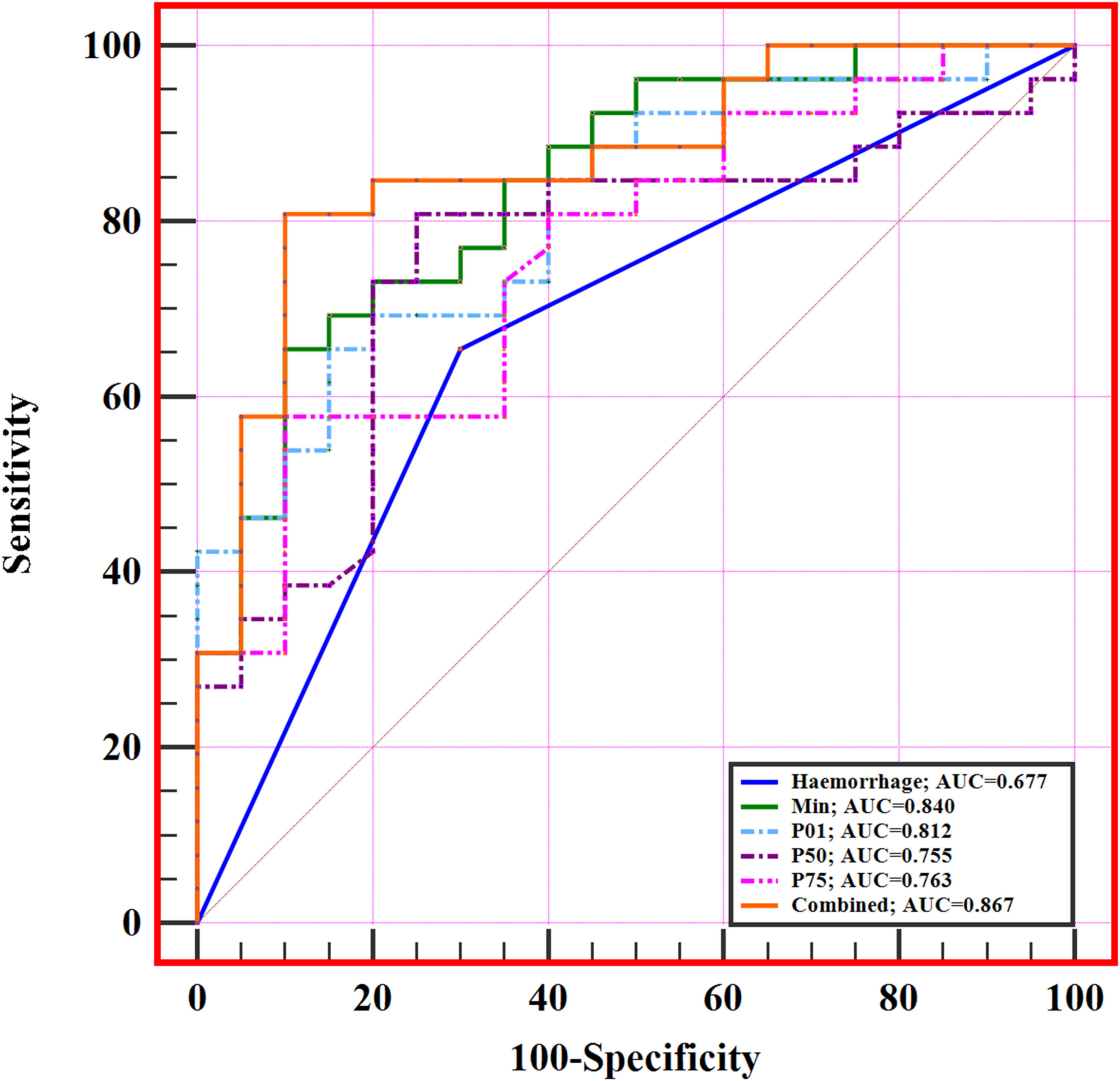
ROC curves of significant MRI morphological features and histogram parameters in predicting Ki-67 expression level in IDH-wildtype glioblastoma. The combined variable (Combined, haemorrhage+min) achieved the highest AUC of 0.867.

The diagnostic performance of significant MRI morphological features and histogram parameters in distinguishing between low- and high-level expression of Ki-67 in patients with IDH-wildtype glioblastoma is presented in **Table 3** and **Figure 4**. The results of the ROC analysis showed that the hemorrhage, min, P01, P50, and P75 could predict the Ki-67 expression level of IDH-wildtype glioblastoma before operation. The combined variable integrating MRI morphological features and histogram parameters (Combined, hemorrhage+min) achieved the highest AUC of 0.867. Delong’s test showed significant differences in AUC between the Combined and MRI morphological features (haemorrhage) (*P*=0.0046). However, there was no significant difference in the AUC between the Combined and histogram parameters (all *P*>0.05).

**Table 3:** Diagnostic performance in distinguishing low- and high-level expression of Ki-67 in IDH-wildtype glioblastoma.

Parameters	AUC (95% CI)	Yoden index	Cutt-off	Sensitivity (%)	Specificity (%)	Accuracy (%)
Haemorrhage	0.677 (0.523, 0.807)	0.35	–	65.38	70.00	67.39
Min	0.840 (0.703, 0.932)	0.55	154.00	65.38	90.00	76.09
P01	0.812 (0.669, 0.912)	0.50	184.00	65.38	85.00	73.91
P50	0.755 (0.606, 0.869)	0.56	390.00	80.77	75.00	78.26
P75	0.763 (0.614, 0.875)	0.48	622.00	57.69	90.00	71.74
Combined	0.867 (0.735, 0.949)	0.71	0.56	80.77	90.00	84.78

AUC, area under the receiver operating characteristic curve; CI, confidence interval. Combined, the combination of MRI morphological features (haemorrhage) and histogram parameters (min).

### 3.5 Correlations between significant histogram parameters and the Ki-67 expression level

**Figure 5:**
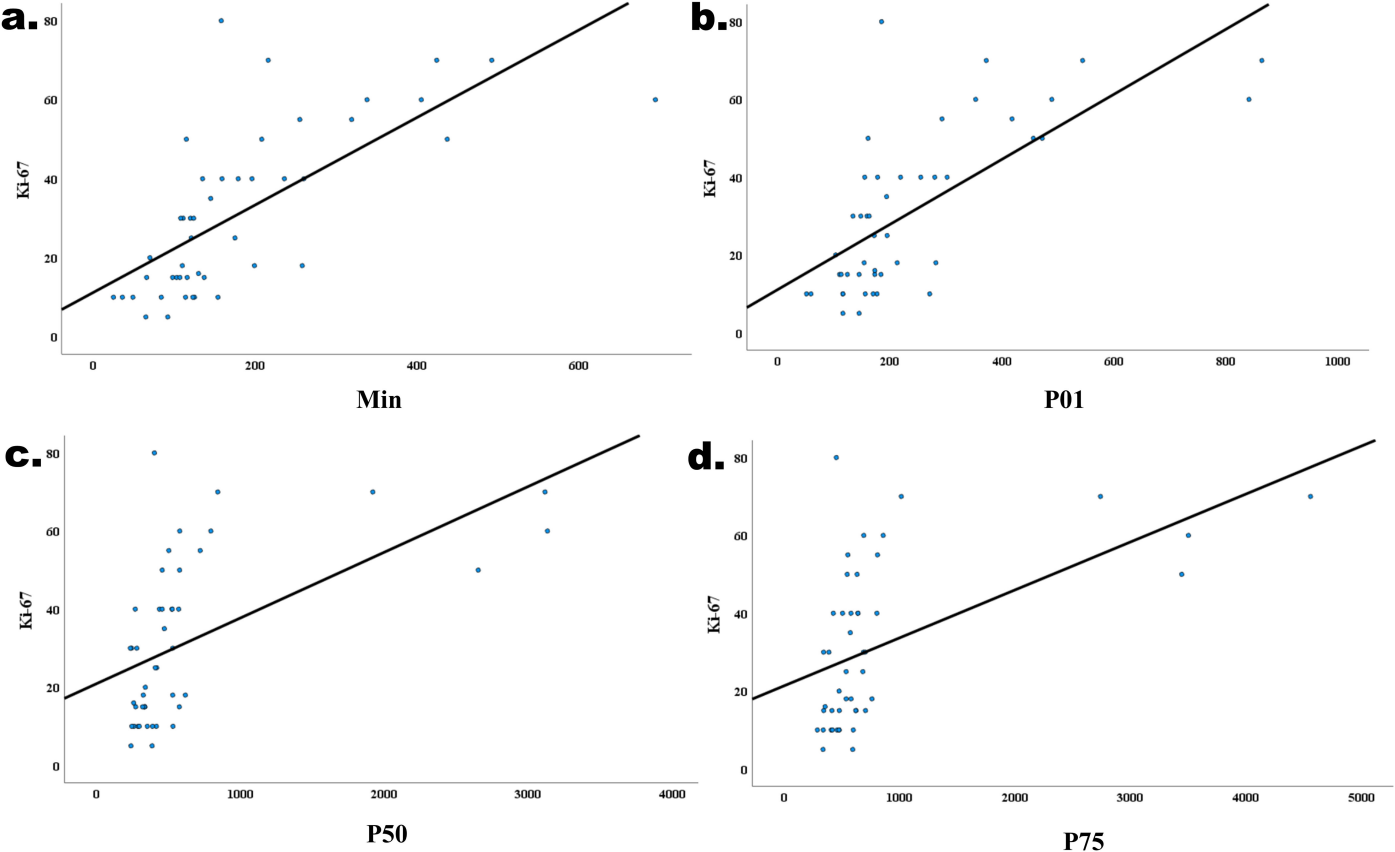
Correlations between the min (*r*=0.680, *P*<0.001, **(a)** P01 (*r*=0.604, *P*<0.001, **(b)** P50 (*r*=0.604, *P*<0.001, **(c)** P75 (*r*=0.604, *P*<0.001, **(d)** and the Ki-67 expression level in IDH−wildtype glioblastoma.

The correlations between the significant histogram parameters and the Ki-67 expression level are presented in **Table 4**. A significant positive correlation was observed between the min (*r*=0.774), P01 (*r*=0.729), P50 (*r*=0.625), P75 (*r*=0.591), and Ki-67 expression level (*P*<0.05, **Figure 5**).

**Table 4:** Correlation between significant histogram parameters and the Ki-67 expression in IDH-wildtype glioblastoma.

Parameters	Ki-67 expression	
	*r*	*P*
Min	0.774	<0.001
P01	0.729	<0.001
P50	0.625	<0.001
P75	0.591	<0.001

*r*=Spearman’s correlation coefficient.

## 4 Discussion

In this study, we investigated the value of MRI morphological features and histogram analysis in the noninvasive prediction of Ki-67 expression level in IDH-wildtype glioblastoma. The results showed that both MRI morphological features and histogram parameters were effective in predicting the Ki-67 expression level in patients with IDH-wildtype glioblastoma. The diagnostic performance can be further improved by combining MRI morphological and histogram features. To our knowledge, this is the first study to assess and compare the effectiveness of MRI morphological features and histogram analysis in noninvasively predicting Ki-67 expression levels in patients with IDH-wildtype glioblastoma.

As a non-invasive examination method, MRI examination is widely used in the diagnosis and evaluation of various brain tumors. Several MRI morphological features have been proven to be useful in predicting the Ki-67 expression levels in patients with IDH-wildtype glioblastoma (8, 18). In our study, we found that haemorrhage was more likely to occur in the Ki-67 high-level expression group, compared to the Ki-67 low-level expression group. This finding is consistent with previous reports in the literature (7, 8). The increased incidence of haemorrhage may be attributed to the high proliferative activity of tumors with high Ki-67 expression levels. These tumors actively proliferate, resulting in numerous new blood vessels with thinner walls, making them more prone to rupture and haemorrhage. When we used the presence of haemorrhage to differentiate between the two tumor groups, the AUC was 0.677. However, unlike what has been reported in the literature (9), no differences in maximum tumor diameter were observed between the low- and high-level expression of Ki-67 in IDH-wildtype glioblastoma in our study. The reason for this is difficult to explain and may be caused by the relatively small sample size in the current study.

Contrast-enhanced T1-weighted images are a routine scan sequence in the evaluation of brain tumors, which could provide information about the blood supply while fully displaying the internal features of the lesion (16, 17). Compared with other advanced MRI imaging sequences, contrast-enhanced T1-weighted image scans are widely performed at all levels of hospitals, especially in primary care institutions (17). However, qualitative analysis of contrast-enhanced T1-weighted images is relatively subjective and cannot fully utilize the deep information included in contrast-enhanced T1-weighted images (14, 16, 17). Histogram analysis of contrast-enhanced T1-weighted images can quantify the heterogeneity of tumors with high utility and reproducibility, making it a promising computer-aided diagnostic method (16–18). Liu et al. (14) found that contrast-enhanced T1-weighted image histogram parameters help predict the grade and proliferative activity of adult intracranial ependymomas. Xue C et al. (13) succeeded in predicting the expression level of tumor-infiltrating CD8+ T cells in patients with glioblastoma preoperatively using contrast-enhanced T1-weighted images histogram analysis. Moreover, histogram analysis is easily operated, with low technical requirements for the operator. It is more likely to be mastered by doctors at all levels of hospitals, and it has excellent clinical applicability and spreadability.

In this study, we performed a histogram analysis of IDH-wildtype glioblastoma. After comparing the contrast-enhanced T1-weighted image histogram parameters between the high-level and the low-level expression group, we found that the min, P01, P50, and P75 of IDH-wildtype glioblastoma in Ki-67 high-level expression group were higher than those of the Ki-67 low-level expression group, and each parameter was able to differentiate between the two groups of tumors. Meanwhile, we further analyzed the correlation between contrast-enhanced T1-weighted image histogram parameters and Ki-67 expression level in IDH-wildtype glioblastoma, and the results showed that the min, P01, P50, and 75 of contrast-enhanced T1-weighted image histogram parameters were positively correlated with Ki-67 expression level. The min and percentile values of the contrast-enhanced T1-weighted image histogram parameters are quantitative reflections of the blood supply within the tumor (11, 13). When Ki-67 is expressed at high levels, the tumor cells are actively proliferating, with abundant neoangiogenesis, and the tumor cells require a richer blood supply to sustain tumor growth, thus exhibiting higher values of the min and percentile values (11, 12). Liu et al. (14) showed that ependymomas with high proliferative activity exhibited higher values of min and percentiles compared to those with low proliferative activity, which is in line with our study. During ROC analysis, we found that the min yielded the best predictive performance, which may be explained by the fact that the min reflects the areas within the IDH-wildtype glioblastoma tumor tissues with the richest blood supply, and the most active growth of the tumor cells, and therefore the difference in the min between the two groups of tumors was also the most significant. The same result was observed in a previous study using contrast-enhanced T1-weighted image histogram parameters to predict the O(6)-methylguanine-DNA methyltransferase promoter methylation status of IDH-wildtype glioblastoma (11). Furthermore, during the correlation analysis, this study also found that the strongest correlation was observed between the min and Ki-67 expression levels. Given the above considerations, we suggest that min may have optimal efficacy in assessing the biological behavior of IDH-wildtype glioblastoma. However, further studies are needed to verify our hypothesis.

Standard deviation, variance, coefficient of variation, skewness, kurtosis, and entropy are quantitative reflections of the distribution of the histogram parameters, indicating the complexity and heterogeneity of the internal tissue components of the tumor. A larger value of these parameters indicates that the internal components of the tumor tissues are more complex and non-uniform, indicating that the tumor has stronger heterogeneity (18–21). Theoretically, IDH-wildtype glioblastoma with a high expression level of Ki-67 has a high degree of tumor cell proliferation and a stronger heterogeneity, which leads to different tumor regions with different proliferative activity, resulting in more complex histological components in the tumor, thus showing a higher numerical value of these histogram parameters. However, no difference was observed in these histogram parameters between the two groups in this study. The reason for this is hard to explain and may be related to the relatively small sample size of this study.

In this study, we compared the diagnostic performance of MRI morphological features and histogram parameters in predicting Ki-67 expression levels in patients with IDH-wildtype glioblastoma. Our findings indicated that both MRI morphological features and histogram parameters could differentiate between the two tumor groups. However, the diagnostic AUC and diagnostic accuracy of the histogram parameters were superior to those of the MRI morphological parameters. This suggests that objective, quantitative histogram parameters are more effective than MRI morphological features in noninvasively predicting Ki-67 expression levels in patients with IDH-wildtype glioblastoma. Furthermore, an exciting observation of this study was that the combined variables integrating MRI morphological features and histogram parameters had an optimal diagnostic performance. In a previous study regarding the pathological grading of brain tumors, the investigators also found that the combined model incorporating MRI morphological features and histogram parameters had significantly better diagnostic performance than either the single MRI morphological features or histogram parameters (22). This may suggest that the combined use of MRI morphological features and histogram parameters has more potential in the evaluation of brain tumors. In other words, in clinical settings with limited access to advanced radiomic tools, preoperative noninvasive prediction of Ki-67 expression levels in IDH-wildtype glioblastoma can be effectively performed by combining conventional MRI morphological features with histogram parameters. This approach leverages the complementary strengths of the two types of features: MRI morphological features, which have been widely used in routine brain tumor assessment (9), and histogram parameters that provide more biologically interpretable compared to complex higher-order radiomic features. While comprehensive radiomics analysis requires specialized expertise, histogram analysis is simpler and easier to master, which offers practical advantages for widespread clinical adoption, particularly in resource-limited settings (11). The synergistic combination of these routinely available imaging biomarkers improves the clinical feasibility of predicting Ki-67 expression levels while maintaining diagnostic reliability.

This study has a few limitations. Firstly, as a single-center retrospective study, the sample size of this study was relatively small, which may reduce the generalizability of the findings. Secondly, this study lacked an external validation set to validate the results. Finally, to speed up the histogram analysis process, only the largest tumor slice was analyzed, which inevitably lost some information and may overlook spatial heterogeneity. In the future, the sample size will be expanded by combining multiple centers and an automated segmentation algorithm will be used for tumor outlining.

## 5 Conclusion

In conclusion, this study demonstrated that both MRI morphological features and histogram parameters could predict the Ki-67 expression level in IDH-wildtype glioblastoma, and the diagnostic performance can be further improved by combining MRI morphological features and histogram parameters, which can provide a quantitative reference for the comprehensive assessment of the patients with IDH-wildtype glioblastoma.

## Data Availability

The raw data supporting the conclusions of this article will be made available by the authors, without undue reservation.

## References

[1] Lapointe S Perry A Butowski NA. Primary brain tumours in adults. Lancet. (2018) 392:432–46. doi: 10.1016/S0140-6736(18)30990-5 30060998

[2] Louis DN Perry A Wesseling P Brat DJ Cree IA Figarella-Branger D. The 2021 WHO classification of tumors of the central nervous system: a summary. Neuro Oncol. (2021) 23:1231–51. doi: 10.1093/neuonc/noab106 PMC8328013 34185076

[3] Price M Ballard C Benedetti J Neff C Cioffi G Waite KA. CBTRUS statistical report: primary brain and other central nervous system tumors diagnosed in the United States in 2017-2021. Neuro Oncol. (2024) 26:vi1–vi85. doi: 10.1093/neuonc/noae145 39371035 PMC11456825

[4] Sipos TC Attila K Kocsis L Bălașa A Chinezu R Baróti BÁ. Clinicopathological parameters and immunohistochemical profiles in correlation with MRI characteristics in glioblastomas. Int J Mol Sci. (2024) 25:13043. doi: 10.3390/ijms252313043 39684754 PMC11642654

[5] Ni J Zhang H Yang Q Fan X Xu J Sun J. Machine-learning and radiomics-based preoperative prediction of Ki-67 expression in glioma using MRI data. Acad Radiol. (2024) 31:3397–405. doi: 10.1016/j.acra.2024.02.009 38458887

[6] Sprenger F da Silva Junior EB Ramina R Cavalcanti MS Martins SB Cerqueira MA. Ki67 index correlates with tumoral volumetry and 5-ALA residual fluorescence in glioblastoma. World Neurosurg. (2024) 189:e230–7. doi: 10.1016/j.wneu.2024.06.023 38857868

[7] Armocida D Frati A Salvati M Santoro A Pesce A. Is Ki-67 index overexpression in IDH wild type glioblastoma a predictor of shorter Progression Free survival? A clinical and Molecular analytic investigation. Clin Neurol Neurosurg. (2020) 198:106126. doi: 10.1016/j.clineuro.2020.106126 32861131

[8] Henker C Kriesen T Schneider B Glass Ä Scherer M Langner S. Correlation of Ki-67 index with volumetric segmentation and its value as a prognostic marker in glioblastoma. World Neurosurg. (2019) 125:e1093–103. doi: 10.1016/j.wneu.2019.02.006 30790732

[9] Bai L Jiang J Zhou J. Assessment of Ki-67 expression levels in IDH-wildtype glioblastoma using logistic regression modelling of VASARI features. Neurosurg Rev. (2023) 47:20. doi: 10.1007/s10143-023-02258-z 38135816

[10] Zhu X He Y Wang M Shu Y Lai X Gan C. Intratumoral and peritumoral multiparametric MRI-based radiomics signature for preoperative prediction of ki-67 proliferation status in glioblastoma: A two-center study. Acad Radiol. (2024) 31:1560–71. doi: 10.1016/j.acra.2023.09.010 37865602

[11] Liu X Han T Wang Y Liu H Zhao Z Deng J. T1 pre- and post-contrast delta histogram parameters in predicting the grade of meningioma and their relationship to Ki-67 proliferation index. Acad Radiol. (2024) 31:4185–95. doi: 10.1016/j.acra.2024.04.005 38653597

[12] Liu H Zhu C Wang X Chen X Li Z Xian J. Prediction of pathological complete response in locally advanced head and neck squamous cell carcinoma treated with neoadjuvant chemo-immunotherapy using volumetric multisequence MRI histogram analysis. Neuroradiology. (2024) 66:919–29. doi: 10.1007/s00234-024-03339-6 38503986

[13] Xue C Zhou Q Zhang P Zhang B Sun Q Li S. MRI histogram analysis of tumor-infiltrating CD8+ T cell levels in patients with glioblastoma. NeuroImage Clin. (2023) 37:103353. doi: 10.1016/j.nicl.2023.103353 36812768 PMC9958466

[14] Liu X Han T Wang Y Liu H Sun Q Xue C. Whole-tumor histogram analysis of postcontrast T1-weighted and apparent diffusion coefficient in predicting the grade and proliferative activity of adult intracranial ependymomas. Neuroradiology. (2024) 66:531–41. doi: 10.1007/s00234-024-03319-w 38400953

[15] Su Y Cheng R Guo J Zhang M Wang J Ji H. Differentiation of glioma and solitary brain metastasis: a multi-parameter magnetic resonance imaging study using histogram analysis. BMC Cancer. (2024) 24:805. doi: 10.1186/s12885-024-12571-5 38969990 PMC11225204

[16] Zhang B Zhou F Zhou Q Xue C Ke X Zhang P. Whole-tumor histogram analysis of multi-parametric MRI for differentiating brain metastases histological subtypes in lung cancers: relationship with the Ki-67 proliferation index. Neurosurg Rev. (2023) 46:218. doi: 10.1007/s10143-023-02129-7 37659040

[17] Liu X Han T Wang Y Liu H Zhou J. Prediction of O(6)-methylguanine-DNA methyltransferase promoter methylation status in IDH-wildtype glioblastoma using MRI histogram analysis. Neurosurg Rev. (2024) 47:285. doi: 10.1007/s10143-024-02522-w 38907038

[18] Gihr GA Horvath-Rizea D Kohlhof-Meinecke P Ganslandt O Henkes H Richter C. Histogram profiling of postcontrast T1-weighted MRI gives valuable insights into tumor biology and enables prediction of growth kinetics and prognosis in meningiomas. Transl Oncol. (2018) 11:957–61. doi: 10.1016/j.tranon.2018.05.009 PMC6008484 29909365

[19] Li J Chen C Fu R Zhang Y Fan Y Xu J. Texture analysis of T1-weighted contrast-enhanced magnetic resonance imaging potentially predicts outcomes of patients with non-wingless-type/non-sonic hedgehog medulloblastoma. World Neurosurg. (2020) 137:e27–33. doi: 10.1016/j.wneu.2019.09.142 31589984

[20] Zhang B Zhou Q Xue C Ke X Zhang P Han T. Nomogram of magnetic resonance imaging (MRI) histogram analysis to predict telomerase reverse transcriptase promoter mutation status in glioblastoma. Quant Imaging Med Surg. (2024) 14:4840–54. doi: 10.21037/qims-24-71 PMC11250314 39022283

[21] Zhao Z Zhang J Yuan S Zhang H Yin H Wang G. The value of whole tumor apparent diffusion coefficient histogram parameters in predicting meningiomas progesterone receptor expression. Neurosurg Rev. (2024) 47:235. doi: 10.1007/s10143-024-02482-1 38795181

[22] Han T Long C Liu X Jing M Zhang Y Deng L. Differential diagnosis of atypical and anaplastic meningiomas based on conventional MRI features and ADC histogram parameters using a logistic regression model nomogram. Neurosurg Rev. (2023) 46:245. doi: 10.1007/s10143-023-02155-5 37718326

